# Research Progress on Hygroscopic Agents for Atmospheric Water Harvesting Systems

**DOI:** 10.3390/ma17030722

**Published:** 2024-02-02

**Authors:** Qi Bai, Wanlai Zhou, Wenzhong Cui, Zhiyong Qi

**Affiliations:** 1 School of Mechanical Engineering, Chengdu University, Chengdu 610059, China; baiq_99@163.com (Q.B.); cuiwenzhong@stu.cdu.edu.cn (W.C.); 2 Institute of Urban Agriculture, Chinese Academy of Agricultural Sciences, Chengdu 610213, China; zhouwanlai@caas.cn

**Keywords:** atmospheric water harvesting, compound hygroscopic agent, design and preparation, adsorption and desorption, porous systems

## Abstract

Adsorptive atmospheric water harvesting systems (AWHs) represent an innovative approach to collecting freshwater resources from the atmosphere, with a hygroscopic agent at their core. This method has garnered significant attention due to its broad applicability, strong recycling capacity, and sustainability. It is being positioned as a key technology to address global freshwater scarcity. The core agent’s hygroscopic properties play a crucial role in determining the performance of the AWHs. This article provides a comprehensive review of the latest advancements in hygroscopic agents, including their adsorption mechanisms and classifications. This study of hygroscopic agents analyzes the performance and characteristics of relevant porous material composite polymer composites and plant composites. It also evaluates the design and preparation of these materials. Aiming at the problems of low moisture adsorption and desorption difficulty of the hygroscopic agent, the factors affecting the water vapor adsorption performance and the method of enhancing the hygroscopic performance of the material are summarized and put forward. For the effect of hygroscopic agents on the volume of water catchment devices, the difference in density before and after hygroscopicity is proposed as part of the evaluation criteria. Moisture absorption per unit volume is added as a performance evaluation criterion to assess the effect of hygroscopic agents on the volume of water collection equipment. The article identifies areas that require further research and development for moisture absorbers, exploring their potential applications in other fields and anticipating the future development direction and opportunities of moisture-absorbing materials. The goal is to promote the early realization of adsorptive atmospheric water harvesting technology for large-scale industrial applications.

## 1. Introduction

“Water is the driving force of all nature”, as Leonardo da Vinci once observed, highlighting its fundamental role in sustaining life. The scarcity of freshwater resources is becoming a global crisis and is even being viewed as a matter of global security [1]. Approximately two-thirds of the world’s population (about 4 billion people) experience severe water scarcity for at least one month each year, according to estimates [2]. Worldwide population growth and rapid economic, agricultural, and industrial development have led to a significant increase in the demand for freshwater resources, exacerbating the issue of water scarcity [3,4]. To address the shortage of freshwater resources, many countries and regions have constructed large reservoirs, implemented off-site water transfers, and desalinated water through large-scale projects. However, these solutions often require extensive construction, long implementation periods, high operating costs, and substantial energy consumption, especially in the case of desalination. Additionally, these solutions are geographically limited. Some countries have implemented intermittent water supply, water mixing, and bulk water distribution as temporary solutions to drinking water needs [5], but intermittent water supply has pressure variations that can affect the life of pipes, and intermittent water supply and water blending increase the likelihood of deterioration in water quality, while bulk water distribution requires consideration of disinfectant residues and microbiological contamination, and is also more costly. Therefore, there is an urgent need to explore supplementary small-scale water intake systems.

Atmospheric water harvesting systems (AWHs) demonstrate significant potential for implementation in arid and impoverished regions among small-scale water abstraction systems [6]. These systems can facilitate portable water production and provide emergency water supplies in the aftermath of disasters [7,8,9]. The United Nations’ Millennium Development Goals (MDGs) underscore the potential of such systems to achieve freshwater self-sufficiency in developing and impoverished regions worldwide [10]. Water vapor, an intermediate state of water, is ubiquitously and perpetually present in the atmosphere. With a total atmospheric content exceeding 12.9 × 10^12^ m^3^, which is approximately six times that of surface river water [11], atmospheric water vapor represents a resource capable of addressing the global freshwater crisis [12]. Historically, research has primarily focused on capturing dew and fog from the air. While fog capture has been successfully implemented in certain regions, this method is applicable to a limited number of areas and requires ambient relative humidity (RH) close to 100% [13,14,15,16,17,18]. Consequently, the development of atmospheric water harvesting systems (AWHs) with broad RH applicability has emerged as a research priority.

Air water harvesting technology is primarily categorized into three methods: direct condensation, membrane separation, and adsorption [19]. Adsorption-based air water harvesting technology leverages the adsorption properties of adsorbent materials on water vapor to enrich the water vapor content, improving the efficiency of water extraction and ensuring a certain water extraction capacity even under low humidity conditions. This makes it a focal point of current research. The hygroscopic agent, which is the core of the adsorption water extraction system, requires energy only during the desorption process, resulting in low energy consumption. Furthermore, it can utilize renewable energy sources such as solar energy, industrial waste heat, and other low-temperature heat sources to realize low-energy water extraction. With the continuous development of adsorption materials and advancements in solar photothermal technology [20], adsorption atmospheric water harvesting technology has emerged as an effective solution to address the scarcity of freshwater resources in deserts, islands, and other regions [19]. It also aids in forming a water cycle in specific environments such as greenhouses, thereby realizing water conservation in production.

**Figure 1:**
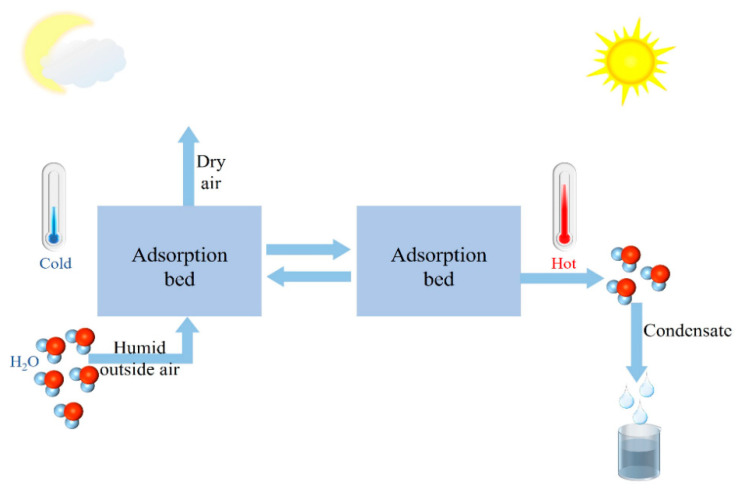
Schematic diagram of adsorption type atmospheric water collection system.

An adsorptive atmospheric water harvesting system captures moisture from the air using a hygroscopic agent at a lower temperature. This system enriches water vapor through temperature rise and desorption, then liquefies the water vapor and collects it, thereby realizing air-to-water extraction [21]. Figure 1 shows the schematic diagram of an adsorptive atmospheric water harvesting system. Consequently, the adsorption and heat conduction properties of the hygroscopic agent significantly influence the water extraction efficiency of the atmospheric water harvesting system [22]. Ideally, the collection system should be efficient, affordable, scalable, broadband, and sufficiently stable [1]. The ideal hygroscopic agent should possess high water absorption, low desorption temperature, high cyclic stability, fast adsorption-desorption rate, simple preparation, and be green and low-cost.

Several articles have reviewed atmospheric water harvesting (AWH) methods [1,23,24,25,26,27]. Beysens et al. [25] introduced various methods such as condensation, fog collection, dew collection, desiccant adsorption, desorption, etc. Tu et al. [23] summarized various atmospheric water harvesting methods in terms of testing devices, performance analysis, simulation methods, etc. Kogan et al. [24] focused on the development and theory of dew collection. However, previous reviews have not paid enough attention to the moisture-absorbing materials themselves in adsorptive atmospheric water harvesting technology. Bilal, Zhou, and Ejeian [28,29,30] preferred devices, applications, designs, etc., and discussed only some of the more hygroscopic moisture-absorbing materials. Bilal et al. [30] reviewed various AWH technologies, their current status, outlooks, and challenges. The article introduced the system design and moisture-absorbing agents, which promoted the development of adsorption AWH technology. However, the discussion on hygroscopic agents mainly focuses on some hygroscopic agents, such as MOFs, and some performance influencing factors of hygroscopic agents, such as thermal conductivity. The analysis of overall hygroscopic agents is lacking. Zhou et al. [28] elaborated the basic requirements and design principles of moisture adsorbents, reviewed the structural design of moisture adsorbents, and promoted the development of moisture adsorbent materials. However, the description of overall moisture-adsorbent materials is not comprehensive enough. Unlike the previous two authors, Ejeian et al. [29] briefly describe physical and chemical adsorbents and mainly review the design of device systems and their applications. The article also discusses the impact of device design on the effectiveness of water harvesting, including energy and cooling. Tu et al. [1]. describe a variety of AWH methods, provide a detailed overview of device system design, and evaluate the hindrances that limit the application of AWH. Thavalengal et al. [31] discuss the principles, advantages, limitations, and potential applications of different AWH technologies. Li et al. [32] and Huan et al. [33] reviewed some categories of moisture-absorbing materials, which did not cover different types of materials and could not fully show the differences between each type of moisture-absorbing materials.

This research review aims to comprehensively overview the types of moisture adsorption materials, their performance characteristics, and moisture adsorption principles. It evaluates each type of moisture adsorption material, takes into account its multiple properties, derives a performance distribution map based on the properties, and then considers the most promising materials in this field. The review also summarizes the factors affecting the adsorption performance and the methods to improve it in order to provide references to the development of moisture adsorption materials in the future. Moreover, the density difference before and after adsorption is proposed as another criterion for evaluating the performance of hygroscopic agents. It also foresees the potential of adsorption atmospheric water harvesting technology in other fields, which could provide more opportunities for the application of this technology in the future. These research studies are also not single but are a part of atmospheric water harvesting (AWH) to promote the development of this technology.

## 2. Adsorption Mechanisms and Isotherms

The mechanism of water vapor adsorption involves the interaction of water molecules with the surface of the adsorbent. This interaction manifests in various ways, including physical adsorption, chemical adsorption, cohesive adsorption, capillary condensation, and surface active sites [34,35,36,37]:

1. Physical adsorption: Physical adsorption is the process by which water molecules attach to the surface of an adsorbent via van der Waals forces. This process is reversible, allowing the adsorbent to release the adsorbed water molecules. However, the amount of water that can be physically adsorbed is relatively small. Porous materials, such as activated carbon, zeolite, and silica gel, typically exhibit this type of adsorption. The larger pore space and specific surface area make it possible to adsorb more adsorbent substances under the intermolecular force. However, the amount of adsorption is still less compared to other adsorption mechanisms, but the adsorption rate is faster. Zhao et al. [38] prepared a composite hygroscopic agent and found that the difference in the adsorption mechanism between salt and zeolite resulted in different adsorption rates for different hygroscopic agents. Additionally, the intermolecular forces between some porous materials, such as zeolite and water molecules, are stronger, and the resolution temperature is higher.
2. Chemisorption: Chemisorption refers to the formation of chemical bonds between water molecules and the surface of the adsorbent. For example, water molecules can form salt hydrates with salts such as calcium chloride (CaCl_2_). The strength of these chemical bonds surpasses that of intermolecular forces, making chemisorption stronger than physical adsorption. Consequently, the energy required to release the water molecules is higher, rendering the process less reversible.
3. Condensed matter adsorption: Under conditions of high pressure or low temperature, water molecules may condense on the surface of the adsorbent to form droplets or water films. However, this adsorption process is generally difficult to occur because it requires a specific temperature or pressure range.
4. Capillary condensation: Capillary condensation refers to the phenomenon where condensation occurs within a capillary tube due to the combined effect of capillary action and the condensation process. When water vapor is present inside the capillary tube, and the temperature of the tube falls below the saturation temperature of the water vapor, the vapor condenses into a liquid state. For instance, the P(SA+AA)-SPH and SPHC-5 gels prepared by Mittal et al. [39] have an extremely high density of interconnected pores that form capillary condensation, resulting in extremely high water vapor adsorption.
5. Surface active sites: Specific molecules or atoms, such as amino groups and hydroxyl groups, which possess high adsorption or catalytic capacity, may be present on the surface of the adsorbent. For example, polymers encompass a wide range of hydrophilic groups, and their adsorption behavior can be influenced by modulating these active sites. Han et al. [40] loaded polypyrrole in mesoporous silica, which increased the number of hydrogen bonds with water vapor and synergized with LiCl to improve the moisture absorption of the material.

In summary, water vapor adsorption is a complex process that involves various mechanisms, including physical and chemical interactions. The factors influencing adsorption on adsorbents are multifaceted. A comprehensive understanding of the adsorption mechanism aids in further elucidating the underlying principles and laws governing this phenomenon, thereby facilitating the optimization of adsorbent design and application and enhancing the performance of the adsorbent.

**Figure 2:**
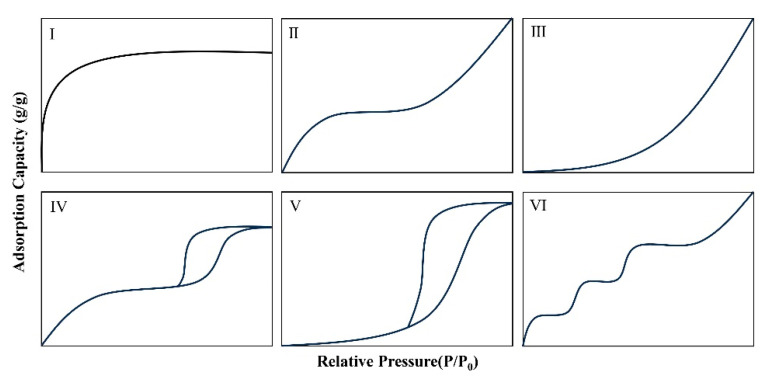
Six types of adsorption isotherms are summarized by the International Union of Pure and Applied Chemistry (IUPAC) [41].

An adsorption isotherm is a relational curve that describes the adsorption behavior of an adsorbate on an adsorbent at a constant temperature. It represents the relationship between relative pressure and the amount adsorbed. In the context of water vapor adsorption, it characterizes the relational curve of adsorption with relative humidity at a specific temperature. Therefore, an isotherm reflects the hygroscopic properties of hygroscopic agents. The International Union of Pure and Applied Chemistry (IUPAC) has summarized six isothermal adsorption types, which correspond to different adsorption situations, as shown in Figure 2 [41]. Type I indicates adsorption on microporous materials; Type II and Type III both show adsorption on macroporous adsorbents, with the difference that Type II has a stronger interaction between adsorbate and adsorbent than Type III; Type IV shows adsorption on a monolayer with capillary condensation; Type V shows adsorption on a multilayer with capillary condensation; and Type VI shows adsorption on multilayer adsorption on non-porous adsorbent with uniform surface.

The hydrophilic properties of the corresponding adsorbent materials differ among the six types. Types I, II, IV, and VI belong to hydrophilic materials, which have a high water absorption rate at low relative humidity. Types III and V isotherms belong to hydrophobic materials, which exhibit weaker hydrophilic properties at lower relative humidity. Figure 1 shows that these six types of isotherms of hygroscopic agents have issues such as low humidity water absorption or narrow hygroscopic range. Therefore, the ideal hygroscopic agent should have high hygroscopicity at low temperatures, high desorption rate at high temperatures, and low equilibrium adsorption amount [1]. In practical applications, the appropriate hygroscopic agent should be selected based on the environment, and the applicable devices should be determined based on the environment and material properties.

## 3. Moisture Absorber Classification and Performance

**Figure 3:**
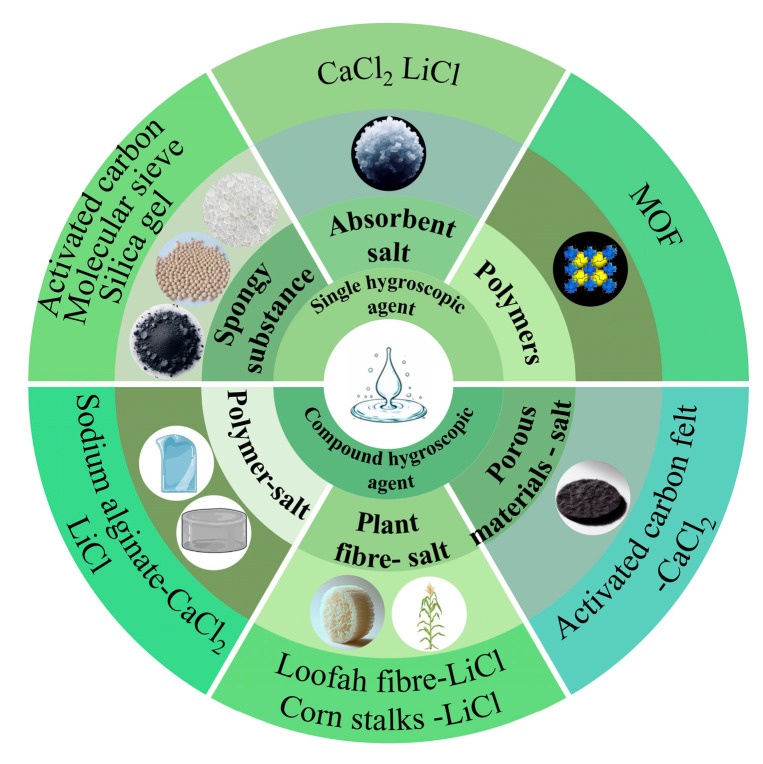
Analysis of the status of moisture-absorbing materials.

Moisture-absorbing materials are mainly classified into two categories: single and composite hygroscopic agents, and the classification diagram is shown in Figure 3. Single hygroscopic agents are mainly classified into three categories: (1) porous materials, such as activated carbon, zeolite, silica gel, etc.; (2) hygroscopic salts, such as CaCl_2_, lithium chloride (LiCl), magnesium sulfate (MgSO_4_), calcium citrate (CHCa_3_O), etc.; (3) hygroscopic polymers, such as MOFs. Composite hygroscopic agents are derived from two or more materials, resulting in a wide variety of types with differing effects. Examples include natural plant material-salt complexes and polymer-salt complexes. Natural plant fibers and bio-gel polymers, in particular, offer the advantages of being resourceful, non-polluting, and possessing good adsorption properties. This makes the composite hygroscopic materials prepared from them, and salts exhibit high potential in atmospheric water harvesting technology. Some of the performance parameters of the hygroscopic materials discussed in the article are presented in Table 1.

### 3.1. Single Hygroscopic Agent

#### 3.1.1. Porous Materials

Zeolite is a crystalline, hydrated, aluminosilicate mineral with aluminum metal sites as water adsorption centers [42] with a three-dimensional framework structure [43], which is a readily available, natural or synthetic porous material, and it is widely used in a variety of industrial applications such as adsorption, catalysis and other applications [44]. Zeolite is characterized by its uniform pore size, large specific surface area, high heat resistance, and broad environmental adaptability. However, it has a high activation temperature and weaker adsorption capacity compared to silica gel. The adsorption capacity of zeolite is inversely correlated with the silica-alumina ratio, meaning that a higher silica-alumina ratio results in weaker water absorption capacity. Zhao et al. [45] found that the 13X zeolite itself had a water absorption capacity of 0.21 g/g at 25 °C and RH = 50% when it was compounded with LiCl. Trapani et al. [46] found that each night, the A3-type zeolites absorbed a large amount of water (25 °C, RH = 40%, moisture absorption 70 mg/g), but it was not resolved during the day. The strong intermolecular forces formed between zeolite and water molecules lead to stable properties after water absorption and high-resolution temperatures, e.g., the resolution temperature of type 13X zeolite reaches 200 °C. Activated carbon is a porous material with a high specific surface area, well-developed pore volume, better heat resistance [47], and abundant chemical groups on the surface. It is inexpensive, nontoxic, odorless, and stable in nature and is commonly used for adsorption and drying. However, its large size, low mechanical strength, and average adsorption capacity make it unsuitable for use as a hygroscopic material. Classical porous solid adsorbent materials, such as zeolite and activated carbon, pose challenges for use in atmospheric water harvesting (AWH). Their adsorption principle is generally physical adsorption, which results in similar water vapor adsorption properties and high desorption temperatures. This makes it difficult for them to be resolved by a low-grade heat source, such as solar energy.

#### 3.1.2. Hygroscopic Salts

Hygroscopic salts achieve adsorption mainly by chemical reaction with water vapor and, therefore, have excellent water absorption properties. The process of water absorption is a chemical process [48], and the hydration process takes place in the form of a solid-solid phase transition [49]. Based on the metal cation, salts can be classified into categories such as calcium, lithium, magnesium, and copper salts. Currently, calcium and lithium salts are more commonly used, with CaCl_2_ and LiCl being the most prevalent. These salts exhibit excellent water-absorbing properties and are abundant in reserves.

Metal inorganic salts can absorb more than 90% of their weight of water in the hydration reaction. Calcium salts, in particular, can absorb several times the weight of water. However, calcium salts are prone to crystallization during the water absorption process. The agglomeration of salt particles reduces gas permeability, which in turn hinders further adsorption of water vapor. Water absorption experiments involving calcium salt generally exhibit high water absorption performance. However, it also has drawbacks, such as requiring high-resolution conditions, exhibiting high corrosivity for some metal materials, and even posing some safety risks. Taken together, calcium salt-based hygroscopic materials still have huge development space [50,51]. LiCl and lithium bromide (LiBr) are commonly used in lithium salt hygroscopic agents [52]. LiCl has the strongest performance among the hygroscopic salts, but like calcium salts, both have similar drawbacks. LiCl is susceptible to deliquescence, agglomeration and clumping, corrosiveness, and swelling during the process of adsorption and resolution [53,54,55]. In summary, hygroscopic salts have the potential to be used in the production of freshwater [48] but to solve the above problems. Hygroscopic salts are generally not used alone but by compounding them with activated carbon and silica gel porous materials to form a composite hygroscopic agent based on porous materials [56,57,58].

#### 3.1.3. Moisture-Absorbing Materials for MOFs

MOFs and their composites have developed rapidly in recent years, and their applications have been mainly focused on gas storage, catalysis, environmental remediation, and energy conversion over the past decades [59,60,61] and the use of MOFs as hygroscopic materials has only been proposed recently [12,62]. MOFs are coordination polymers with regular pore channels, adjustable pore sizes, high porosities, varied topologies, and high specific surface areas [63,64], which are porous coordination compounds with an open framework structure formed by inorganic metal centers and organic ligands connected by coordination bonds. Currently, there are various types of MOF materials, and the main ones in AWH are the UiO series, MIL series, aluminum-based, zirconium-based series, ZIF series, etc. There are three kinds of water adsorption principles of MOFs [64]: (i) physical adsorption, (ii) chemical adsorption on metal sites, and (iii) capillary condensation. The water adsorption mechanism surfaces its water adsorption performance is mostly related to the pore structure, thanks to the excellent pore structure of MOFs, which makes this kind of material have the characteristics of large moisture adsorption capacity and fast rate of moisture adsorption rate.

The diversity of MOF components allows for targeted design through rational compositions. A combination of different metal and organic ligands can improve the pore structure, adsorption sites, and other factors that determine the adsorption performance of MOFs, thereby improving the adsorption capacity or achieving the selectivity of adsorption. Kim et al. [12] human-synthesized MOF-801 in the simulation of a natural environment with a low heat of 1 kW/m^2^ for 24 consecutive hours to collect water in a continuous cycle with a daily water extraction of about 2.8 L/kg. Yilmaz et al. [65] enhanced the adsorption kinetics of the MOF by activation and achieved a water extraction of 6.39 g/g at RH = 90%. It can be seen that the strong hygroscopic properties of MOFs make them promising for hygroscopic applications, but most of them are unstable, expensive, and even have toxicity [62]; whether they can be applied in water harvesting systems must be considered in terms of their stability and toxicity.

### 3.2. Compound Hygroscopic Agent

A single hygroscopic agent contains only one hygroscopic component, and its singular composition leads to unbalanced performance. It may excel in certain aspects but fails to meet the requirements of the atmospheric water harvesting (AWH) system for efficient water collection. As a result, composite hygroscopic agents have gradually become the primary focus of research. Composite hygroscopic agents contain a variety of hygroscopic components, each with potentially different adsorption principles, adsorption performance, and mechanical properties. Therefore, composite hygroscopic agents benefit from the combined performance of various components and can be adjusted based on the environmental conditions of their use, thereby expanding their scope of application.

#### 3.2.1. Porous Materials-Salt

**Figure 4:**
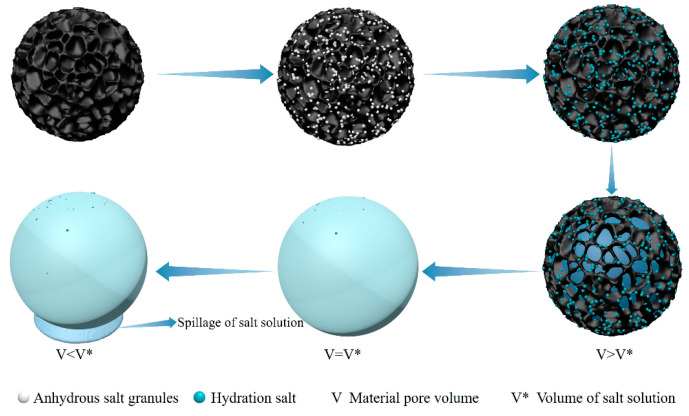
Porous material-salt composite hygroscopic agent hygroscopic principle diagram.

Traditional adsorbent materials such as zeolite and activated carbon are simple and inexpensive to prepare and thus are widely used in various fields [66,67], but the porous materials have poor hygroscopic properties and high-resolution temperatures [46,68]. Hygroscopic salt is prone to problems such as deliquesce, leakage, and agglomeration [53,54,55]. Figure 4 shows the moisture absorption principle diagram of a porous material-salt composite hygroscopic agent. The salt is attached to the inside of the porous material, and the salt adsorption of water vapor is fixed inside the porous material after hydrolysis. This effectively alleviates the drawbacks caused by salt hydrolysis. Salt leakage can occur when the salt content in the composite hygroscopic agent is too high, but this can be avoided by controlling the salt content. The composite hygroscopic agent, formed by combining porous material and salt, effectively improves the defects of the two materials. It retains the high hygroscopicity of hygroscopic salt and the stability of porous material, greatly enhancing the performance of the hygroscopic agent and promoting the innovation and advancement of atmospheric water harvesting technology.

There are three main methods for preparing composite hygroscopic agents: impregnation, grinding and mixing, and curing. In the impregnation method, the porous material is immersed in a salt solution of varying concentrations, followed by a prolonged period of impregnation and subsequent drying. This allows the salt to embed in the porous material, resulting in a fully mixed and uniform composite hygroscopic agent. The grinding and mixing method involves thoroughly grinding and mixing the porous material and salt according to a certain proportion. This method is simpler than the impregnation method [47]. The curing method involves preparing a composite wicking agent of a fixed shape using an adhesive or pressure. This method is more complex to prepare, and the degree of curing is difficult to control. Therefore, the impregnation method has become a popular research topic for modifying hygroscopic agents and developing new composite hygroscopic agents in recent years.

Activated carbon fiber (ACF) is a fibrous material with activated carbon as the main component. Its high specific surface area and porosity make it an important adsorbent material. Several scholars have prepared a variety of composite hygroscopic agents with excellent hygroscopic properties by compounding activated carbon fiber with salts. Entezari et al. [69] used activated carbon mats compounded with three kinds of salts were compared to obtain the best effect of the composite hygroscopic agent of ACFs-LiCl at 25 °C and RH = 70%, the water absorption capacity was 2.9 g/g, and in the designed prototype, the water extraction reached 1.51 g/g (RH = 70%). Wang et al. [58] used impregnation to obtain a series of ACF-CaCl_2_ composite hygroscopic agents with different salt contents, and the hygroscopic capacity of ACF30 at 20 °C and RH = 70% was 1.7 g/g, which is more than three times that of silica gel-CaCl_2_.During the preparation of the ACF-salt composite hygroscopic agent, the direct contact between ACF and salt solution will make ACF softer, which will have an important effect on the adsorbent bed structure [70]. Wang et al. [71] cured activated carbon fibers with silica gel first during the preparation of the ACF-LiCl composite hygroscopic agent, and the cured compounded ACF-LiCl composite hygroscopic agent had the highest water absorption rate of 1.2 g/g and showed excellent resolving and mechanical properties. The resolving amount was 0.6 g/g at 77 °C and RH = 20%, and it was easy to form before drying and difficult to deform after drying, with excellent mechanical structural properties.

Silica gel is an inorganic material with high porosity and a large specific surface area. It has excellent adsorption properties due to the accumulation of silica particles. The good pore structure of silica gel makes it a popular choice as a carrier for composite hygroscopic agents. Liu Yefeng et al. [56] used silica gel compounded with CaCl_2_ to achieve a water absorption rate of 0.5 g/g at 25 °C and RH = 70%, which was greater than that of a single silica gel hygroscopic agent. Yu et al. [68] proposed a hygroscopic agent compounded from silica gel matrix and LiCl and investigated the effect of different configuration parameters on the hygroscopic performance, and the results showed that the different content of LiCl in the composite hygroscopic agent would significantly affect the hygroscopic performance of the composite hygroscopic agent. Simonova et al. [72] used saturated calcium nitrate (Ca(NO_3_)_2_) solution impregnated with silica KSK to synthesize the composite hygroscopic agent SWS-8L, which increased the water absorption rate to 0.2~0.3 g/g, which is 3.5 times higher than that of pure KSK.

Zeolites are a class of microporous crystalline materials, and although they exist in nature, they are now often synthesized to meet the growing industrial demand, including aluminosilicate molecular sieves A (LTA type), X (FAU type), Y (FAU type), and ZSM-5 (MFI type); pure silica silica-1 (MFI type), and Aluminum Phosphate AlPO-n series, among others [73]. Zeolites usually require high-resolution temperatures, but the synthesis of a variety of ordered mesoporous molecular sieves with excellent properties such as ordered pore channels, adjustable pore size, uniform size, and large specific surface area offers the possibility of preparing composite hygroscopic agents [74]. Zhao et al. [45] prepared composite hygroscopic agents with different salt contents using 13X zeolites with LiCl and LiCl were found to be at concentrations ≥ 35%, and in the presence of high humidity conditions, composite hygroscopic agents appeared to be deliquescent, in this salt concentration composite hygroscopic agent has a greater risk of leakage, so the salt concentration should be reduced in the actual preparation, the composite hygroscopic agent prepared using a salt concentration of 30% at 25 °C, RH = 50%, the rate of water absorption is 0.7 g/g, which is three times that of the zeolite itself.

#### 3.2.2. MOFs Composite Hygroscopic Agents

Certain MOFs have permanent porosity with reasonable stability as well as high hygroscopicity [75], so their properties can be further improved by incorporating hygroscopic salts, introducing hydrophilic sites, or modifying or doping the functional groups. Xu et al. [55] successfully prepared LiCl@MIL101(Cr) by restricting hygroscopic salts to MOFs. This composite hygroscopic agent integrated the salt chemical adsorption, deliquescent adsorption, and other adsorption processes to improve its hygroscopic properties, and it is an efficient composite hygroscopic agent with a hygroscopicity of 0.77 g/g at 30 °C and RH = 30%. Luis et al. [76] reported a spray-drying continuous flow synthesis of a new type of solid adsorbent by varying the CaCl_2_ concentration to prepare three UiO-66-based composite hygroscopic agents (CaCl_2_@ UiO-66_38, CaCl_2_@ UiO-66_50, CaCl_2_@ UiO-66_53), which reached 1.93 g/g, 2.24 g/g, and 2.59 g/g, respectively, under the adsorption conditions of 20 °C and RH = 90%. Wu et al. [77], with the help of the excellent photothermal ability of titanium carbide (Ti_3_C_2_), successfully synthesized Ti_3_C_2_-doped UiO-66-NH2 composites and embedded them into sodium alginate (SA) polymer networks with crosslinked structures, which ultimately yielded vertically aligned porous networked TUN/SA composite hygroscopic agents with good shapes. The photothermal conversion ability of the hygroscopic agent is effectively improved by the innovative doping of Ti_3_C_2_. When resolved by sunlight irradiation, it can rapidly resolve 99.3% of the adsorbed water at RH = 20%. The porous network-like structure provides a channel for water molecules to diffuse, further accelerating the adsorption and resolution of water vapor. The excellent adsorption performance demonstrates the feasibility of improving the performance of composite hygroscopic agents by doping and embedding, which provides a new idea for the preparation of new composite hygroscopic agents.

The excellent performance of MOF composite hygroscopic agents makes them a promising candidate for AWHs [78]. However, similar to single MOF hygroscopic agents, their high price, poor hygroscopicity under low humidity conditions, and potential toxicity are factors that hinder their development in the field of water absorption. Therefore, it is crucial to address these shortcomings to enable practical applications and to explore new research directions.

#### 3.2.3. Polymer-Based Composite Hygroscopic Agents

Polymers are compounds composed of many identical or different monomer molecules linked by chemical bonds. Different monomers lead to varying hygroscopic properties. The capillary phenomenon generated by the network structure of the polymer promotes the entry of water vapor. Water vapor enters and forms hydrogen bonds with a large number of hydrophilic functional groups. Some ionic groups ionize, increasing the internal concentration difference, which further promotes the entry of water vapor. As a result, polymer hygroscopic agents exhibit stronger hygroscopic properties. Complexing metal cations in polymers with salts or modified complexes with polymers can enhance the performance of composite hygroscopic agents [79].

**Figure 5:**
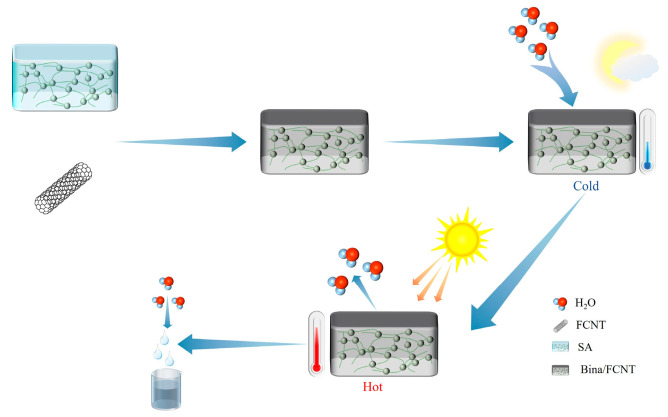
Alginate gel composite hygroscopic agent moisture absorption principle diagram.

Entezari et al. [79] modified sodium alginate (SA) hydrogel with hygroscopic salts LiCl and CaCl_2_ to improve its water absorption properties. They also doped functionalized carbon nanotubes (FCNTs) to improve their photothermal conversion ability. This greatly increased the temperature under solar irradiation and favorably increased the desorption capacity (Figure 5). The produced Bina/FCNT is a very efficient composite hygroscopic agent with a water absorption rate of 5.6 g/g at 25 °C and RH = 70%. However, the mechanical properties of sodium alginate hydrogel in the dry state are reduced and easy to break. A large amount of water absorption may cause loss of stability and recovery difficulties. Overcoming its poor mechanical properties, poor regeneration performance, stability, and other defects is an urgent problem that needs to be studied and solved. Zhao et al. [80] infiltrated the hygroscopic polypyrrole (PPy-Cl) into the polyisopropylacrylamide (poly-NIPAM) A super hygroscopic gel (SMAG) was successfully prepared by infiltrating hygroscopic polypyrrole (PPy-Cl) into poly-NIPAM (poly-NIPAM) network to achieve efficient adsorption and resolution, and the hydrophilic network structure of polyisopropylacrylamide to achieve a large amount of water storage. At 90%, 60%, and 30% RH, the water absorption rate is 6.7 g/g, 3.4 g/g, and 0.7 g/g, respectively, and the wider moisture absorption range and excellent moisture absorption performance greatly expand the scope of application. It remains to be investigated whether the disadvantages of the two polymers, such as temperature sensitivity and limited regeneration ability, have a greater impact on the application of adsorption AWH. Taken together, these polymers provide an effective and novel method for water vapor adsorption.

#### 3.2.4. Plant Fiber–Salt Composite Hygroscopic Agents

Natural plant fibers are generally non-polluting, renewable, and abundant in resources. Some plant fibers possess excellent pore structures, providing numerous sites for salt attachment and a multitude of channels for water vapor diffusion and transport. Surface hygroscopic salt adsorbs water vapor through various types of channels, transporting water to the interior of the hygroscopic agent. The abundance of these channels results in very high adsorption efficiency. During water evaporation, numerous pores facilitate the diffusion of water vapor to the exterior of the hygroscopic agent, positively impacting desorption.

**Figure 6:**
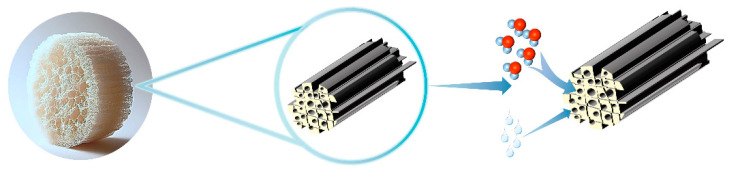
Principle of moisture absorption of loofah composite hygroscopic agent.

Yao et al. [81] used LiCl to fill the hollow three-dimensional network of loofah fibers to enhance the hygroscopic properties of the material. The loofah fibers with porous three-dimensional networks, hierarchical macropores, and microchannels lead to their higher water vapor adsorption and volatilization efficiency. After LiCl adsorption of a large amount of water vapor converted to liquid water, the liquid water is gradually infiltrated into the internal pores of the loofah through its porous structure and can be stored through the bacterial cellulose (BC) and carbon nanotubes (CNT) hydrophilic network formed by BC/CNT for storage. Thus, it has high moisture absorption and water storage capacity (Figure 6). BC is a polysaccharide with a nanofibrous structure [82]. At the same time, cellulose undergoes attenuation of mechanical properties with increasing humidity [83], replacing loofah skin with BC and CNT gel-like substance to form a loofah-like structure, and adhesion between the materials increases with increasing humidity, and the composite of multiple materials enhances the mechanical properties of the materials. The CNTs on the surface of the material have extremely high light absorption ability, which can enhance the light-heat conversion ability, and can reach more than 60 °C under the light intensity of 1.3 kW/m^2^, which makes the water vapor generation increase, and its hollow channels can promote the diffusion of water vapor outward, which can further improve the resolving ability of the material. The participation of LiCl and CNTs made the material’s moisture-absorption performance improve dramatically, and the dried LBC@LiCl reached 0.81~2.47 g/g of hygroscopic capacity after 300 min at 40~90% RH. 1.94 g/g and 2.65 g/g of LiCl were achieved after 9 h at 80% and 90% RH, respectively. Gong et al. [84] prepared hygroscopic materials for hygroscopicity and power generation by using corn stover, which, as an agricultural waste, is widely available in various environments. It favorably promotes its application in remote, arid, and power-deficient areas. The homogeneous channels of corn stover and the uniform distribution of LiCl enable it to have 0.5~1.8 g/g under the environmental conditions of RH of 20~80%, and it can resolve 90% of the water under the sunlight; meanwhile, using the modification of carbon ink, the modified hygroscopic material can generate 0.6 V, which has the potential of power supply for adsorption devices. From the present point of view, the use of agricultural waste has enough advantages to be used for the preparation of high-performance hygroscopic materials, and in the process of hygroscopicity has the potential to generate electricity, the use of agricultural waste to prepare hygroscopic materials can not only be used to solve the problem of water scarcity but also is a potential way to deal with agricultural waste to reduce agricultural pollution, which helps to maximize the use of resources.

**Table 1:** Classification and properties of moisture-absorbing materials.

Material Classification	Absorbent	Hygroscopic Conditions	Maximum Moisture Absorption (g/g)	Desorption Conditions	Literature Sources
Spongy substance	13X zeolite	25 °C, RH = 50%	0.21	—	[45]
	A3 zeolite	25 °C, RH = 40%	0.07	Not desorbed below 60 °C	[46]
Absorbent salt	LiCl	25 °C, RH = 70%	2.5	Not desorbed below 80 °C	[79]
	CaCl_2_	25 °C, RH = 70%	2.0	Not desorbed below 80 °C	[79]
MOFs	MOF-801	25 °C, RH = 20%	0.25	—	[12]
	PC-MOF	25 °C, RH = 90%	6.39	23 °C~65 °C	[65]
Porous materials-salt	ACF-LiCl	25 °C, RH = 70%	2.9	80 °C	[69]
	ACF-CaCl_2_	20 °C, RH = 70%	1.7	—	[58]
	Cured ACF-LiCl	25 °C, P0/P = 0.9	1.2	77 °C, RH = 20%	[71]
	Silica gel-CaCl_2_	25 °C, RH = 70%	0.5	—	[56]
	13X zeolite-LiCl	25 °C, RH = 50%	0.7	—	[45]
MOFs-salt	LiCl@MIL101(Cr)	30 °C, RH = 30%	0.77	76 °C~97 °C	[55]
	CaCl2@ UiO-66_53	20 °C, RH = 90%	2.59	370 K	[76]
	TUN-2/SA	25 °C, RH = 40%	0.23	100.2 °C	[77]
Polymers	Bina/FCNT	25 °C, RH = 70%	5.6	80 °C	[79]
	SMAG	10~40 °C, RH = 90%	6.7	40 °C~63 °C	[80]
Vegetable fiber–salt	LBC@LiCl	25 °C, RH = 40~90%	0.81~2.47	Bulk desorption at 60 °C	[81]
	BCS	RH = 20~80%	0.5~1.8	Solar 1 kW/m2 resolution 90%	[84]

### 3.3. Performance Evaluation Criteria

**Figure 7:**
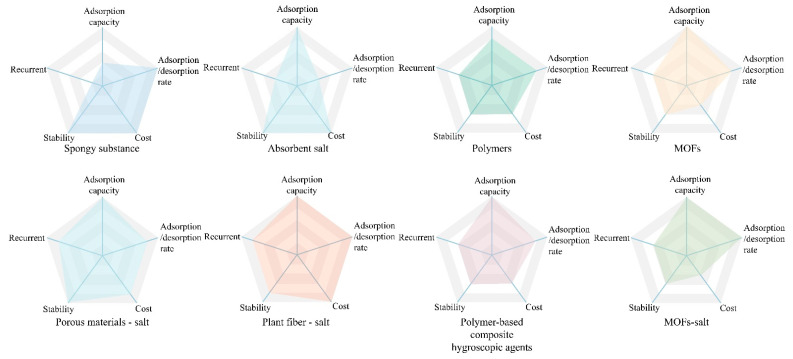
Comparison of the performance characteristics of various types of hygroscopic agents.

Figure 7 is a performance characteristic diagram that summarizes the performance and evaluation criteria of various hygroscopic agents. It is evident that different hygroscopic agents exhibit different performance characteristics and are, therefore, suitable for different environmental conditions. Plant fiber-salt, porous material-salt, and polymer-composite hygroscopic agents in composite hygroscopic agents in AWH systems exhibit great potential.

However, current research focuses on the amount of moisture absorbed per unit mass as the main parameter for evaluating moisture absorption performance [54,81,85,86,87]. In actual use, volume is an important factor limiting the number of devices and materials used, so the amount of moisture adsorbed per unit volume of moisture-absorbing materials should also be used as one of the performance evaluation criteria, and this is related to the density of the material after adsorption. The increase of moisture after adsorption, the density of the material should increase, but considering the expansion of the material after moisture adsorption, its density may not change significantly, so the higher the difference between the density of the material after adsorption and before adsorption, means that the material has a higher amount of moisture adsorption with a smaller volume, which is more meaningful for the practical application of moisture adsorbents. Equation (1) is the density difference before and after adsorption:

(1) $$\Delta \rho =\rho -\rho_{0}$$

*ρ* and *ρ*_0_ are the densities of the material after and before adsorption, respectively.

## 4. Factors Affecting and Improving Adsorption Performance

### 4.1. Influencing Factors

Atmospheric water harvesting is affected by various factors, such as the device, hygroscopic agent, and weather. The hygroscopic agent is the key component of adsorptive atmospheric water harvesting, directly involved in the cycle of water vapor adsorption and desorption, and determines energy consumption [88,89]. The adsorption performance of the hygroscopic agent has a direct impact on the effectiveness of atmospheric water harvesting. A comprehensive study of the factors that influence adsorption performance is essential for the rational design, selection, improvement, and optimization of the adsorption process of adsorbents. The primary factors that affect water vapor adsorption are:

1. Pore structure: The adsorption performance is significantly influenced by the pore structure type, specific surface area, pore diameter, and other pore structure properties of the adsorbent. Various types of pore structures affect the diffusion and transport of adsorbent molecules inside the adsorbent. A uniform pore structure is conducive to molecular diffusion. For example, carbon nanotubes doped in Bina/FCNT are conducive to maintaining the homogeneity and stability of the porous network structure, which provides a channel for the diffusion and transport of water vapor [79]. Pore volume refers to the volume size of the pores inside the adsorbent, which determines the adsorption capacity under specific conditions. Therefore, it is essential to study and enhance the adsorbent pore structure to improve the adsorption performance of the adsorbent.
2. Surface properties: The surface properties of adsorbents, such as surface polarity, chemical composition, functional groups, and contact angle, directly influence the interaction between the adsorbent and adsorbate molecules, as well as adsorption selectivity, which in turn affects the amount and rate of adsorption. Surface functional groups are the most important surface properties that affect adsorption performance. The type, concentration, and spatial distribution of these functional groups significantly affect the adsorption behavior of water molecules on the adsorbent.
3. Activation conditions: activation conditions have an important impact on the hygroscopic agent, such as zeolite molecular sieve, activated carbon, and other activation temperatures are high, after adsorption of water vapor in the sun irradiation is difficult to desorption, so the activation of the difficult conditions of the material used in the sun desorption of adsorption of the water collection system is difficult.
4. Temperature: Temperature has a significant impact on the adsorption performance of adsorbents in two key areas: the adsorption process and the preparation of the adsorbent. During the adsorption process, temperature primarily affects the thermal motion of water molecules and the stability of clusters. It also influences the binding capacity of functional groups and water molecules, thereby affecting both the adsorption capacity and rate. In the context of adsorbent preparation, temperature induces changes in functional groups, surface charge, and contact angle, which in turn leads to significant modifications in the adsorption properties [90].
5. Humidity: Changes in humidity cause shifts in the position of the adsorption equilibrium. As humidity increases, the adsorbent is more likely to reach its adsorption saturation point, resulting in a higher adsorption quantity and rate. Conversely, lower humidity levels correspond to a reduced adsorption capacity of the adsorbent.
6. Photothermal conversion and heat transfer: The sorbent’s photothermal conversion and heat transfer capabilities can increase the material’s temperature when exposed to sunlight or other heat sources, thereby facilitating desorption. The increase in desorption promotes an increase in the number of adsorption cycles and, thus, an increase in the amount of water withdrawn. Therefore, heat and mass transfer capabilities are also crucial for hygroscopic agents.
7. Water stability: In the process of moisture absorption, moisture absorbent will inevitably be in the environment containing a large amount of water. High water stability is the inevitable requirement of moisture absorbent [91]. High stability can maintain the pore structure and other properties of the hygroscopic agent, which is conducive to maintaining efficient hygroscopic performance and structural design.

### 4.2. Enhancement Methods

Improving the adsorption characteristics of materials is a critical goal in the field of adsorbent research and application, especially for the development and utilization of adsorptive atmospheric water harvesting systems. The following are common strategies used to enhance adsorption performance:

1. Improvement of adsorbent pore structure: (1) Wet activation, i.e., the material is soaked and etched by liquid medicines to make the pore channels open and the specific surface area increase; (2) High-temperature treatment, which can make the material’s specific surface area, pore volume, etc. change by high-temperature treatment; (3) Material synthesis using template or template-free method to synthesize materials with specific structure or morphology, e.g., Marchesini et al. [92] synthesized a BN with a specific surface area of more than 1900 m^2^/g by the template-free method; (4) Composite, Zhao et al. [80] prepared a super-hygroscopic gel with uniform pore size by compositing.
2. Surface modification: Using modification, changing the surface properties of the adsorbent, increasing functional groups, hydrophilic particles, and other hydrophilic groups can improve the adsorption performance, such as Entezari et al. [79] prepared gels with CaCl_2_, LiCl modification, so that the sodium ions in the sodium alginate gels and the exchange of more hydrophilic Ca^2+^, Li^+^, and thus improve the adsorption performance.
3. Material composite: Composite adsorbents formed by combining two or more materials using specific methods can leverage the benefits of different adsorbents and effectively enhance the adsorption performance of the adsorbent.
4. Photothermal conversion and heat transfer: The photothermal conversion and heat transfer capacity of the composite hygroscopic agent can be enhanced by adding materials with higher photothermal conversion and heat transfer capacity, increasing the desorption temperature of the material and improving the water collection efficiency. For instance, Entezari et al. [79] developed hygroscopic gels doped with carbon nanotubes that have a very high photothermal conversion capacity. In simulated solar experiments at 1 kW/m^2^, the material temperatures exceeded 70 °C, which increased the rate of water release.
5. Adsorbent regeneration: To increase the actual amount of water produced, it is necessary to improve the adsorption and resolution conditions through the adsorption device and increase the number of cycles.

## 5. Future Directions

Currently, freshwater scarcity and water pollution are increasingly severe worldwide. Efficiently obtaining water resources is a crucial step towards achieving sustainable development. The atmosphere contains abundant and stable water vapor, which is an important freshwater resource. Rational utilization of atmospheric water resources can effectively address the issue of freshwater scarcity.

The atmospheric water harvesting system based on hygroscopic materials has great potential in solving the problem of water scarcity. Its hygroscopic properties can also be used for humidity control in the fields of agriculture and industry. It is expected to achieve a local water cycle based on humidity control to help water-saving industries, such as using this technology to control the humidity of greenhouses and recycle water to achieve the purpose of stabilizing humidity and conserving water. Therefore, the research and development of new and efficient moisture-absorbing materials is the main task and the core of the current work. In order to promote the early realization of large-scale application of atmospheric water harvesting technology, more resources should be invested in the development of the next generation of smart wicking materials, and future research can be strengthened in the following areas.

1. Currently, most composites of porous materials, salts, and polymers are produced through impregnation, which is a time-consuming and inefficient process. Investigating the effects of simpler methods, such as milling, on composite adsorbent materials may be advantageous.
2. Inorganic hygroscopic salts such as CaCl_2_ and LiCl, which are commonly used today, have strong hygroscopic properties. However, they are prone to deliquescence in high humidity environments and can corrode adsorption equipment. Therefore, exploring the impact of weakly corrosive organic acid salts in composite hygroscopic agents has become a significant trend.
3. Designing hygroscopic agents that are customized to specific conditions based on the application environment can optimize their performance.
4. Designing energy-efficient water collection equipment that matches the performance of the hygroscopic agent and the usage environment can increase the number of adsorption/desorption cycles of the material through the water collection device, thereby maximizing the amount of water produced.
5. Instead of limiting the amount of water vapor adsorbed per unit mass as an evaluation criterion in the past, the difference in the density of the material before and after moisture adsorption is used as part of the criterion for evaluating moisture adsorption performance, which in turn takes into account the effect of moisture-absorbing materials on the volume of the water-collecting device.
6. This technology can be used for water recycling in agriculture and industry, not just for drinking water. The choice of moisture absorption materials and devices should be based on the water quality requirements.
7. In the development of efficient hygroscopic agents, it is essential to conduct research on the long-term stability of materials, economy, sustainability, and the complexity of the synthesis process.

## 6. Conclusions

Atmospheric water is a potential source of freshwater, and its rational use can increase the diversity of freshwater sources [93]. Diverse water sources ensure the sustainability of water resources and reduce dependence on a single source. Adsorption atmospheric water harvesting technology is characterized by wide applicability, low energy consumption, good sustainability, and low cost, and has significant advantages in acquiring water resources. Adsorption of atmospheric water harvesting technology can improve the utilization of renewable energy, alleviate energy waste and water shortage, and promote energy technology and economic development, in line with the concept of sustainable development. The lack of efficient hygroscopic agents is the main problem limiting this technology, and research on hygroscopic agents will help water vapor become an economical source of daily drinking water. In this paper, we review and summarize recent research on AWH hygroscopic agents, leading to the following primary conclusions:

1. Adsorption AWH is a simple, compact, adaptable, scalable, economical, low-energy, renewable, and clean water extraction technology with significant potential for application.
2. Currently, the large-scale application of adsorption AWH is challenging. Performance indicators such as efficiency, economy, adaptability, cycling, and stability are not simultaneously satisfied, making the research and development of high-performance, balanced moisture-absorbing materials a current research focus. Performance indicators are not limited to moisture absorption per unit mass but also consider moisture absorption per unit volume.
3. The study of moisture-absorbing materials should shift from single to composite materials, with a focus on utilizing low-cost light and heat conversion technologies or materials.
4. Plant fibers are a natural material that has great potential for reducing costs and increasing environmental sustainability.
5. AWH technology is not limited to providing access to potable water but can also be extended to industrial water conservation, facility-based agricultural water recycling, and agricultural irrigation.
6. Moisture-absorbing materials should not be limited to fixed temperature and humidity. Instead, materials should be developed to adapt to different conditions depending on the environment in which they are used, maximizing material performance in the corresponding environment.
7. The device and hygroscopic materials are inseparable. Microcontrollers and other control systems can be used to regulate sunlight exposure, which can increase the number of adsorption and desorption cycles and, thus, the actual amount of water pumped.
8. Water extraction efficiency can be improved by using moisture-absorbing materials, optimizing system design, and enhancing condensation.

This paper presents an overview of trends and innovations in adsorbent AWH hygroscopic agents and proposes innovative applications of adsorbent AWH technology, with hygroscopic agents as a core component. This area should be a top priority for research, development, and use.

## Data Availability

Not Applicable.
